# Use of live chat in higher education to support self-regulated help seeking behaviours: a comparison of online and blended learner perspectives

**DOI:** 10.1186/s41239-021-00253-2

**Published:** 2021-04-06

**Authors:** Jaclyn Broadbent, Jason Lodge

**Affiliations:** 1 School of Psychology, Deakin University, Geelong, Australia; 2 Centre for Research in Assessment and Digital Learning, Deakin University, Geelong, Australia; 3 School of Education, The University of Queensland, St Lucia, QLD Australia

**Keywords:** Help-seeking, Live chat, Instant messaging, Online learning, Blended learning, Higher education, Self-regulated learning

## Abstract

Help-seeking is an essential self-regulatory and metacognitive skill. In the online learning environment, much of the learning is self-directed and there are generally less opportunities to receive help in real time. The uses of email and discussion boards are the most common method of seeking help in these environments. The current study explored students’ perceptions of the use of live chat technology for online academic help-seeking within higher education, with a focus on comparing online and blended learners’ perspectives. Participants were 246 students who were studying psychology online (*n* = 91) or in blended learning (*n* = 155) environments. The live chat technology was well received by both groups, especially for its ability to provide instant, real-time, and convenient help. Live chat was particularly well received by online learners, who were more satisfied, felt more cared about by the teaching team, and would be more likely to recommend it to others than blended learners. Further, online learners reported that live chat gave them better access to staff, and felt that this feature was a good approximation for more traditional face-to-face conversations. As an online, synchronous, private help-seeking tool between student and teacher, these findings provide a strong endorsement for the use of live chat in higher education, particularly for online learners.

## Introduction and review of the literature

With much of the learning in higher education now occurring online, with stay at home restrictions of COVID-19 as an extreme example, there are reduced opportunities for traditional student-to-teacher and student-to-student interactions (Boling et al., 2012). One form of interaction that is potentially under threat is help-seeking, an important self-regulated learning strategy (Schunk & Zimmerman, 2012). Students seek help from teachers and peers for a range of reasons, such as navigation of content and resources, as well as checking understanding, seeking feedback, course advice, or to discuss personal matters (Lodge, 2010). As an online student, a common means of seeking help is through email or use of online discussion boards (Kitsantas & Chow, 2007; Koc & Liu, 2016). These methods do not give online learners the opportunity for synchronous help-seeking. This raises the question of how to support learners with help-seeking behaviours in online learning environments equivalently to those in face-to-face classrooms. This is a timely issue as the number of online students continues to rise annually (Allen & Seaman, 2014). These students, by the very nature of studying remotely, have less opportunity for face-to-face interaction. The emergence of synchronous chat technology, such as live chat, which is designed as a virtual help-desk on online shopping websites, has the potential to allow private conversation between teacher and student in real-time. This opens new possibilities to support academic help-seeking behaviour online.

### Online academic help-seeking

Help-seeking is an essential self-regulatory and metacognitive skill (Schunk & Zimmerman, 2012). The ability to self-regulate one's study-related behaviours and cognitions is linked to important educational attainment outcomes, including academic achievement (Zimmerman, 1990). To be considered 'self-regulated', a learner must be motivated, metacognitively involved, and active in his or her learning process (Zimmerman, 1986, 2015). In reference to Zimmerman's three-phase self-regulated learning (SRL) model (Zimmerman & Moylan, 2009), help-seeking can occur in all three phases: (1) it can occur in the forethought phase when learners are planning action (e.g., seeking help to understand the requirements of an assignment); (2) during the performance phase, the learner takes action, engages with the task and self-corrects during performance (e.g., asking for help with writing an assignment); and (3) in the reflection phase, students may evaluate their performance (e.g., asking for help to understand feedback on an assignment). Currently, much of the literature on help-seeking behaviour in higher education has focused on help-seeking behaviours of traditional students, with only more recent literature focusing on online help-seeking behaviours (e.g., Cheng et al., 2013).

Academic help-seeking behaviour includes a range of decisions made by learners, which includes deciding whether they needs help, deciding who they need help from and what form of help they require (Chyr et al., 2017). Not all learners are willing to seek help. Help-seeking behaviours range depending on the learners attitude, the sources of help they need (e.g. informal or formal), the type of help (e.g. instrumental or executive), and their avoidance behaviours (Karabenick, 2003). In this article, online academic help-seeking is considered here as any behaviour aimed at obtaining assistance from others online (teaching staff, peers, etc.) to help reduce an academic challenge or overcome an impasse. In the online environment, this often includes the use of email, discussion boards in the learning management system (LMS), social media (e.g. WhatsApp, Facebook, Twitter etc.), or question asking in a live online class (Cheng et al., 2013). On the discussion boards, students post questions under discussion topics that are open to a response from teachers or students. Email is similar, except private and is typically one-on-one between the teacher and student. In both cases, conversations with students are usually asynchronistic, and can occur over long periods of time. This may lead to stilted and disjointed conversations, where students wait for a response to a question they would likely prefer immediately (Koc & Liu, 2016). If the student has reached an impasse, this delay halts their learning progression until they receive the help they seek.

Synchronous conversation can occur between online students and teaching staff via telephone or video chat (e.g. Zoom). These modes of communication allow one-on-one or one-to-many interactions. However, reports suggest that phone calls continue to have reduced popularity as a mode of communication as they are seen as disruptive, inconsiderate, and overly time-consuming (Buchanan, 2016; Turner, 2019; Wiest, 2019). Likely indicating that phone calls may not be student preferred method of synchronous chat with teaching staff, particularly those from younger generations. Further, as many Australian Universities run subjects with thousands of enrolments, organising phone or zoom meetings may not be an efficient way for teaching staff to communicate with students one-on-one. While email, phone calls and discussion boards are fit for purpose in some contexts, Koc and Liu (2016) suggest that teaching staff need to be more creative in promoting online help-seeking behaviours by using online apps and mobile technologies to enhance student support. This is particularly important for online learners who have reduced avenues for communication.

### Live chat

Live chat is a synchronistic, help-orientated, communication tool that has been successfully used by shopping websites. Live chat is service-based helpdesk software that allows online chat between the service provider (e.g. teacher) and client (e.g. student; Kang et al., 2015). Live chat is different from instant messenger services as the former allows anyone to chat with the service provider (e.g. teacher) on a host website (e.g. LMS) rather than via social media, a communication service between known people or a service where the customer needs to download the technology to use it (Broadbent, 2020; Broadbent & Lodge, 2020; Matteson et al., 2011). Live chat is designed for one-on-one interactions, rather than peer learning or group chat, and is synchronous only (Kang et al., 2015). That is, communication only occurs when both parties are available.

To the authors' knowledge, there are no studies to date that explore student perceptions of the use of live chat technology within courses in higher education. However, in the past decade, live chat has become popular within university library services (see Matteson et al., 2011), and courses have adopted other, similar technology for support such as instant messaging and social media. Several studies have looked at instant messaging services within a higher education context such as chat rooms (Mtshali et al., 2015), Twitter (Hitchcock & Young, 2016; Luo et al., 2017), Facebook (Amador & Amador, 2017; Vivian, 2011), text messaging (Kay & Lauricella, 2015; Lauricella & Kay, 2013), video chat (Kay & Lauricella, 2015), and WhatsApp (Klein et al., 2018; Pimmer et al., 2018; Raiman et al., 2017; Robles et al., 2019; So, 2016). These studies found that instant messaging increases the number of student-to-student and student-to-teacher interactions (Klein et al. 2018; Mtshali et al., 2015), enhances sense of connection (Klien et al. 2016; McInnerney & Roberts, 2004), provides high levels of student satisfaction (e.g. Luo et al. 2017; Robles et al. 2019), and provides student support (Klien et al., 2016). Furthermore, students like that instant messaging responses are immediate and timely (Lauricella & Kay, 2013).

The majority of these studies looked at the use of instant messaging in group work, which may (e.g. Mtshali et al., 2015) or may not (e.g. Sun et al., 2018) include a teacher and, hence, did not look directly at online academic help-seeking behaviours solely between teacher and student. Given its private nature, text messaging is the most similar to live chat. A study by Lauricella and Kay (2013) found that students liked the instant nature and timeliness of text messaging. Even so, students said reminders about assessments sent out by teaching staff were the main benefit, rather than using it for help-seeking. Conversely, when students text peers, they were more likely to do it for help-seeking reasons. Studies that have looked at live chat in other contexts, such as used by the Library, have shown positive responses. A recent systematic review by Matteson et al. (2011) found that across 59 studies live chat users were positive about their experiences with live chat, and found that live chat allowed for rich conversation, which included the ability to receive instruction through this medium.

### Statement of problem

While there are many similarities between instant messaging and live chat, such as the immediate nature of the chat, there are also differences. Live chat is designed explicitly to allow interaction between the service provider (e.g. teacher) and the customer (e.g. student), is not attached to any social media platform, and does not allow customers (e.g. students) interact with one another (Go & Sundar, 2019; Kang et al., 2015; Matteson et al. 2011). These differences suggest that caution is warranted for generalising the findings from instant messaging and current limited uses of live chat to broader use of live chat in higher education. Accordingly, the merits of live chat as an educational tool remain unclear. Further, none of the studies above directly compare the perceptions of different types of students, such as blended learning students (who have a face-to-face component to their studies) with the needs/perspectives of students who are online-only.

There is reason to speculate that students in these different study modes may behave differently when using the tool. There is some evidence that blended learners, who have the opportunity for face-to-face interaction, may have different perspectives than online-only students in how useful online technology such as live chat may be. Studies that have looked at help-seeking behaviours of on-campus, online and/or blended learning students have found that help-seeking behaviours differ between these groups. For example, online students have been shown to use help-seeking behaviours less than on-campus students (Broadbent & Poon, 2015; Richardson et al., 2012), as well as when directly compared to blended learning students (Broadbent, 2017). Broadbent and Poon (2015) speculated that measures of help-seeking behaviours did not adequately capture online behaviours. Still, it is possible that, as their only mode of communication with teaching staff, online students may differ in their use and attitudes towards live chat technology.

### Aims

The current study aims to explore the use of live chat technology for online academic help-seeking within higher education, with a particular focus on different perceptions of online or blended learning students. In particular, this study explores:

1. Online and blended students’ attitudes towards live chat, the perceived usefulness and their willingness to use live chat technology for academic purposes to communicate with teaching staff in a higher education setting.
2. To determine online and blended students’ preferences across different communication methods such as email, discussion boards, and live chat.

## Method

### Participants

Participants were 246 students currently attending [anonymous] University in Australia, and aged between 18 and 60 years of age. Blended learners (*n* = 155) were more likely to be female (90%) and were undertaking a Bachelor’s degree in Psychology (57%), and had a mean age of 21.68 (SD = 5.93) years. Online learners (*n* = 91) were also more likely to be female (88%), had a mean age of 31.66 (SD = 10.09) years, and currently undertaking a Bachelor’s degree in Psychology (69%). Age differences between groups were significant; *t*(125.70) = 8.546, *p* < 0.001. Gender composition did not significantly differ across groups. All participants were enrolled in one or more of eight psychology subjects spread over years 1–3 of an undergraduate bachelor degree (five subjects were in first year, one subject in second year, and two subjects in third-year) that were using live chat as a help-seeking tool during one or more trimesters. Most participants were taking only one subject using live chat (*n* = 123), followed by two (*n* = 66), four (*n* = 18), or three subjects (*n* = 15). Twenty-four participants did not specify how many subjects they experienced live chat in, though they were retained as their responses indicated exposure to live chat in their studies. Please note too that blended learning students in this study have a face-to-face component to their studies, whereas online students have no face-to-face requirements and all studies are conducted online.

### Materials

#### Live chat platform

**Fig. 1:**
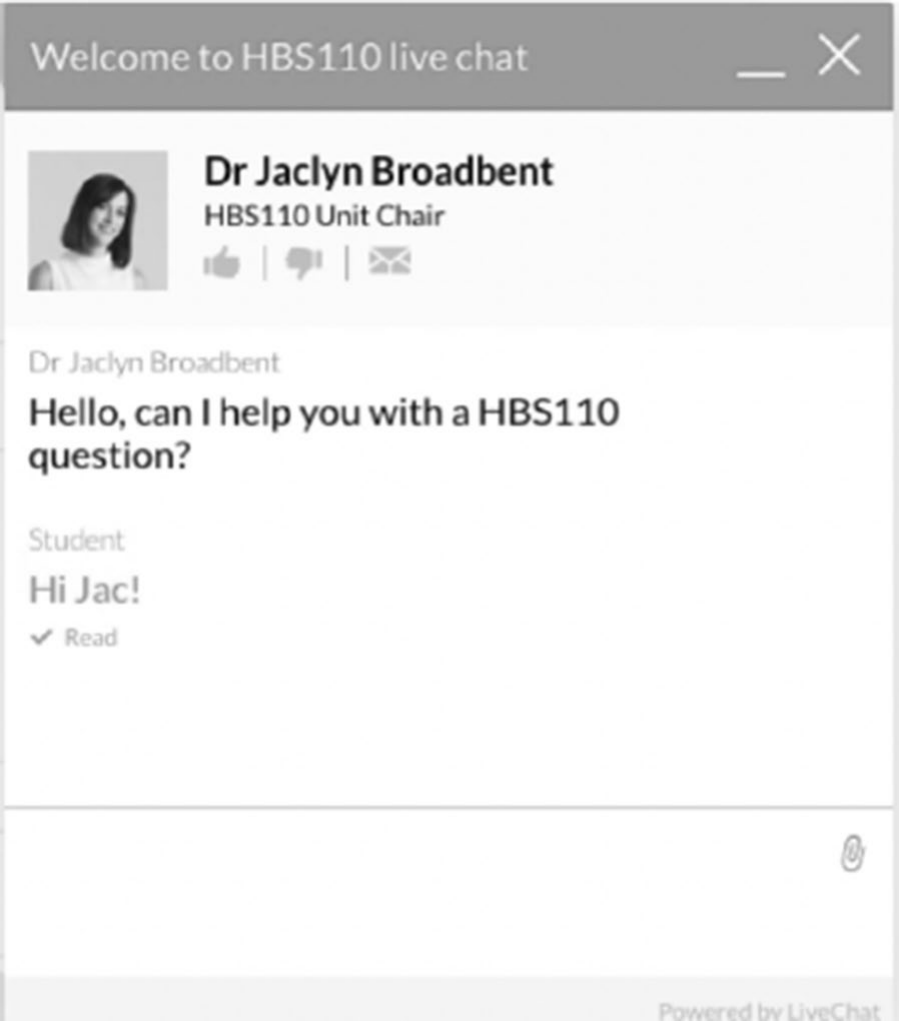
Example of the LiveChat pop-up widget (student view)

The live chat software was developed by LiveChat® (https://www.livechatinc.com). LiveChat® is a service-based helpdesk software that allows online chat between the service provider and client (in this case, teacher and student). Live chat is different from instant messenger services which provide a communication service between friends. The LiveChat® platform allows staff to talk one-on-one with multiple students at a time. The chat is private between teacher and student, such that the chat with one student is not visible to any other student. The platform allows students and staff to share files, save transcripts, and chat in real time. Students can locate the LiveChat® software on each Learning Management Systems (LMS) homepage as a pop-up widget. LiveChat® was monitored by the subject coordinators and by senior tutors, and the widget was only visible to students in a subject when the corresponding subject staff were available. If staff were not available, e.g. outside their virtual office hours, LiveChat was not available to learners. Figure 1 depicts an image of what the learner sees when using the platform if staff are available.

#### Questionnaire

##### Questions about live chat

The questionnaire contained nine open-ended questions, and four questions on a 4-point rating scale (Strongly agree to disagree strongly) plus a did not use/not applicable option. The four rating scale questions asked participants (a) how useful live chat was for ‘just-in-time support’, (b) whether using live chat made them feel like the teaching team cared about supporting their learning, (c) how satisfied they were with live chat as a communication tool, and (d) whether they would recommend live chat to other students.

Open-ended questions asked participants about (a) what questions they asked, (b) what questions they did not want to ask, (c) what they found most useful about live chat, (d) what they liked, and (e) how it could be improved. Participants were then asked about how they seek help and were prompted to give reasons why they would use (a) live chat, (b) email, and (c) discussion boards to seek help.

### Procedure

Ethics was approved by the University’s ethics committee. Participants were enrolled in one or more subjects that were using live chat. Subjects ran in dual mode with blended and online students enrolled together. Live chat was used as the communication channel during virtual office hours. The first author ran one of the subjects that used live chat. Students were alerted to the staff virtual office hours on live chat through announcements on the subject homepage. Each subject used live chat in different ways and at different times of the day that fit with the availability of staff. Most subjects had a set virtual office hours each week. During assessment weeks, additional virtual meeting hours were to facilitate the extra communication needs of students. At the end of the trimester, an announcement on the learning management system alerted students to the study. Data were collected via an online survey provided as a link on announcements on the LMS. Participation was voluntary. Participants went into the draw for 2 × $50 gift vouchers.

### Data analysis

This is a cross-sectional study using mixed methods. An inductive thematic analysis was conducted to draw themes from responses for each question for the group as a whole. Braun and Clarke’s (2006) six-phase method was used: (1) familiarising with the data, (2) initial codes were generated, (3) themes were generated and then reviewed, (4) some themes split into new themes, and those without enough data to support them were collapsed into other themes, (5) themes were named, and (6) a report generated. Outside expertise was consulted to ensure themes made sense, were clearly defined and a subsection of data was cross-coded. After themes were derived for each question, the number of comments for each theme were counted separately for blended learners and online learners. Differences in frequency of themes between groups were measured using chi-square analysis.

## Results

### Data cleaning

Three hundred and sixty-seven participants started the survey. One hundred and one participants were excluded as they did not complete any, or only completed the demographic portion, of the survey. Twenty-five participants completed the survey twice. Participants’ first survey was kept and the second survey excluded if they (a) completed both surveys within the same trimester and named the same subject both times (*n* = 11), or (b) completed both surveys within different trimesters, and there was some overlap of subjects (*n* = 2). From the 25 participants who completed the survey twice, both responses were included if they (a) occurred in different trimesters with different subjects (*n* = 9), or (b) occurred in the same trimesters but with different subjects (*n* = 3). One person was excluded because they were not enrolled in any psychology subjects with live chat. Three participants were excluded for being under the age of 18, and three participants were excluded for giving unreliable data, e.g. humorous responses. A total of 246 participants remained (details in participants section).

### Satisfaction, just-in-time support, cared and supported, and recommend to others.

Both online and blended learners were positive about their experience with live chat, on average strongly agreeing to all statements. *Mann–Whitney U* tests were conducted in order to determine whether online learners differed in their experience of live chat to blended learners. The results show that online learners significantly felt the teaching team cared more about them, were more satisfied and more likely to recommend live chat to others than the blended learners. See Table 1.

**Table 1:** Online and Blended learners use of live chat: satisfaction, just-in-time support, care and support, and recommend to others

		Blended learner (%)	Online learner (%)	
Useful for just-in-time support	Strongly agree	56.3	68.8	*U* = 3950.50 *p* = 0.057
	Agree	37.8	29.9	
	Disagree	1.7	0	
	Strongly disagree	4.2	1.3	
Felt the teaching team cared about supporting their learning	Strongly agree	60.3	82.5	*U* = 3723.50 *p* = 0.001
	Agree	31.4	16.3	
	Disagree	4.1	0	
	Strongly disagree	4.1	1.3	
Satisfaction as a communication tool	Very satisfied	74.8	90.4	*U* = 3524.00 *p* = 0.007
	Satisfied	20.9	9.6	
	Dissatisfied	3.5	0	
	Very dissatisfied	0.9	0	
Recommend to other leaners	Definitely would recommend	64.3	85.9	*U* = 3829.00, *p* = 0.001
	Probably would recommend	29.4	12.8	
	Probably wouldn’t recommend	4	1.3	
	Definitely wouldn’t recommend	2.4	0	

Blended learners n = 144; Online Learners n = 85; excludes N/A

### Thematic analysis

Each question was analysed for themes as a whole, and then the proportion of learners from each group that made a comment that fit the theme was recorded. Chi-square analyses were used to determine if the difference in proportions between groups was significant. Learners’ comments were not included if they said they did not use live chat or responded with n/a or alike. Comments could have more than one theme and so may add up to more than 100%.

#### I asked questions on live chat about…

Table 2 shows that five themes emerged plus a ‘question not specified’ theme regarding what questions learners asked on live chat. The majority of learners asked questions about their assessments. Chi-square analyses show that the themes did not differ significantly. Themes are discussed in more detail below.

**Table 2:** What online and blended learners asked questions about on live chat

I asked questions on live chat about…	Proportion of blended learners *n* = *116*	Proportion of online learners *n* = *73*	Chi-square	*p* value
Assessment	74.14%	74.32%	0.03	0.85
Subject / degree general questions	16.38%	20.27%	0.53	0.47
Subject content	19.83%	14.86%	0.69	0.41
Finding information	11.21%	8.11%	0.44	0.51
Using technology	7.76%	8.11%	0.01	0.91
Question not specified	0.00%	2.70%	3.21	0.07

Regardless of whether blended or online, three-quarters of learners mentioned that the questions they asked on live chat were related to the subject assessment. Some learners stated it helped them clarify aspects they did not understand, for example:

*“…mostly regarding the assignment for the [subject]. It was really helpful because I couldn’t find the results on the discussion board and the assignment was due in the next 2 days. So it was really helpful speaking to the [subject co-ordinator] so I was able to clarify some parts of the assignment and therefore add all of the important information into it.”*

The second largest theme was general subject or degree questions such asking timetabling questions or help with course related issues, for example: “I asked questions mainly about the library in regards to posting book to and from my home town in [XXXX]”.

This was followed by questions that were about content in the subject but not necessarily about assessment, for example: “I would ask questions about what some of the learning objectives mean if I don't understand how they were written.” To a lesser extent students also used the live chat widget to find learning resources or get help with using technology.

#### I did not ask questions on live chat about…

Table 3 shows that six themes emerged plus an ‘other’ theme regarding what questions learners did not ask on live chat. The majority of learners said they were comfortable asking anything on live chat. Chi-square analyses show that the themes did not differ significantly between the groups except questions that were around assessment, in particular extension, feedback, grades and remarking, with significantly more blended learners avoiding questions of this nature on live chat than online learners. Themes are discussed in more detail below.

**Table 3:** What online and blended learners did not asked questions about on live chat

I did not ask questions on live chat about…	Proportion of blended learners *n* = *100*	Proportion of online learners *n* = *55*	Chi-square	*p* value
I was comfortable asking anything	45.00%	50.91%	0.50	0.48
Assessment (e.g. extensions, feedback, grades, remark)	24.00%	7.27%	6.71	**0.01**
Things personal to me	11.00%	14.55%	0.42	0.52
Questions that could or have been answered elsewhere, or would benefit others	9.00%	9.09%	0.00	1.00
In depth or complicated answer needed	4.00%	7.27%	0.78	0.38
Subject content	4.00%	3.64%	0.01	0.91
Other	7.00%	12.73%	1.42	0.23

Bold indiactes significant value (*p* < .05)

About half of the learners said they “felt comfortable asking anything” or that there was “no question I would have not asked”. Approximately one-quarter of the responses from blended learners shows they did not want to ask question related to the formal aspects of an assignment such as requesting an extension, clarifying feedback, asking for a remark, or discussing a grade. For example: “Questions relevant to procedures such as special consideration or getting an assignment re-marked.” However, this was mentioned significantly less often for online students.

A small portion of students felt uncomfortable asking private / personal questions (*n* = 20) e.g. “private questions personal to me….” “Questions which required confidentiality or a timeline”. However, from the comments provided, it seems some students did not know that other students could not see their questions “such as some private question since it is public”.

Interestingly, students discerned what they should ask for an immediate response and what could wait because it was basic or they thought other students would benefit from the answer if it was posed in a public forum such as the discussion boards e.g. “Any that could have benefited the other students by asking on the forums” or “basic questions relating to [subject] content that could easily be asked/answered on the [subject] discussion boards.”

Some students preferred to keep complicated questions for other forums “I would have felt comfortable asking any question on the live chat—providing that it was simple and easy to explain” and “I would avoid to in depth questions and keep it short and sweet”. This was slightly related to subject content, where a few students stated they did not ask questions about it “I was unsure whether to ask to explain a theory or term from the book as that felt more like a seminar thing to do”.

#### I found live chat to most useful…

Table 4 shows that six themes emerged plus an ‘other’ theme regarding the questions learners found most useful live chat. The most mentioned themes were having live chat available around assessment time and that live chat gave an instant response. Chi-square analyses show that the themes did not differ significantly between the groups except for “when I need a quick response” was mentioned significantly more by online learners. Two other themes significantly differed in frequency across groups. Unsurprisingly, online students mentioned using live chat in the evening was most useful and blended learning students mentioned that it is most useful when they were not on campus. As online students are never on campus, it is unsurprising that this was not a theme for them. Themes are discussed in more detail below.

**Table 4:** What online and blended learners found most useful about live chat

I found live chat to most useful…	Proportion of blended learners *n* = *125*	Proportion of online learners *n* = *78*	Chi-square	*p* value
Around assessment time	48.80%	35.90%	3.25	0.07
Whenever I need a quick response just-in-time reply	24.80%	47.44%	11.05	** < 0.01**
During the day / before after class	10.40%	6.41%	0.95	0.33
To clarify resources / find resources	7.20%	5.13%	0.34	0.56
When I am not on campus	4.80%	0.00%	3.86	**0.05**
In the evening	0.80%	5.13%	3.75	**0.05**
Other	10.40%	8.97%	0.11	0.74

Bold indiactes significant value (*p* < .05)

All the themes except one (to clarify resources) pertained to time of use. A large proportion of students found live chat was “…most useful, during the assignment period towards the due date” and “When I was completing assessment tasks, usually week leading up to deadlines.”

A similar number of students said it was useful when they needed a fast response “When I needed an assignment question answered quick while I was working on it and not have to wait several days” and “The fact that it was live and I got answers almost straight away. That’s where the discussion boards can lack because you can wait almost a day for an answer to your question when you need to know right away most times.” This theme also included it being available just-in-time for them, that is, “anytime”, “all the time” and “every time I needed it”. This theme was particularly important for online students who mentioned it significantly more often than blended learning students.

A small amount of both blended and online learners preferred to use live chat during the day or before or after class. Significantly more online learners indicated they preferred the evening and significantly more blended learners preferred using live chat when they are at home.

#### I like live chat because…

Table 5 shows that six themes emerged plus an ‘other’ theme regarding what learners like about live chat. The majority of learners like that it was instant and convenient for them to use. Chi-square analyses show that the themes did not differ significantly between the groups, except on the theme regarding increased access to staff. The replication of face-to-face conversations was mentioned more often by online learners than blended learners as a reason they liked live chat. Themes are discussed in more detail below.

**Table 5:** What online and blended learners liked most about live chat

I like live chat because…	Proportion of blended learners *n* = *120*	Proportion of online learners *n* = *78*	Chi-square	*p* value
Instant / real time / convenient	76.67%	78.21%	0.64	0.80
Access to staff / replicate face-to-face	20.00%	48.72%	18.13	** < 0.01**
Easy to use / good design	18.33%	21.79%	0.36	0.55
Helpful	11.67%	10.26%	0.10	0.76
Personal / cared	5.00%	7.69%	0.60	0.44
Private	4.17%	1.28%	1.34	0.25
Other	2.50%	0.00%	1.98	0.16

Bold indiactes significant value (*p* < .05)

More than three-quarters of learners mentioned that they liked the instant / real time / convenience of using live chat. They felt it was a good use of their time, helped continue with the flow when they were studying, and they liked the real time responses. For example, “I love that it's an instant response i.e. my questions were answered within a minute or two and I could then move on and keep working on my assignment” and “…..the instant nature of it. Emails usually have a lag and for example right before a deadline time is of the essence! Made me feel more relaxed knowing id be more likely to get a response”.

Relationships with and accessibility to staff were also important to learners, particularly online learners, who mentioned this significantly more often than blended learners and in nearly 50% of comments. For example, “……It feels more personal—almost like talking directly to someone, rather than sending an email. It actually makes being an online student feel less isolating. You are aware that being off-campus, that you miss out on one to one contact with tutors and lecturers, but this allows for one to one contact in a different way.”

One fifth of learners mentioned they liked live chat because of how easy it was to use or that its good design features (such as that it popped-up or that it could send transcripts of conversations) made it an easy and useful mode of communication. For example, “easy to use and very familiar with the function (similar to social media sites)”, and “Easy to use, and helpful. Visibility—good that it pops up when you log on”.

Lastly, learners liked how helpful live chat was. For example, “It didn't seem like I was annoying them with questions and it felt a little bit more personal (I announce 'HELP!! I can't make it change to the right thing!!' and [the teacher] responded with 'Oh no! Don't worry it will be ok' and then not only helped me but also waited to make sure it was completed properly.”

#### Improvement I can suggest for live chat are…

Table 6 shows that four themes emerged plus an ‘other’ theme regarding suggested improvements for live chat. The most common theme mentioned was that learners did not think that live chat needed any improvement or that it could be improved by being available more often and in more subjects. Chi-square analyses showed that blended learners were more likely to mention that they thought no improvements were necessary. No other significant differences were found between groups. Themes are discussed in more detail below.

**Table 6:** Suggested improvements made by online and blended learners about live chat

Improvements I can suggest for live chat are…	Proportion of blended learners *n* = *111*	Proportion of online learners *n* = *73*	Chi-square	*p* value
No suggested improvements (excludes n/a or no response)	43.24%	27.03%	4.75	**0.03**
Available more often / more subjects	27.03%	27.03%	0.00	0.96
Design (e.g. anonymous, transfer files, video, drawing, transcript, group chat)	26.13%	22.97%	0.19	0.66
Set times / notification of availability (e.g. advertise times)	7.21%	13.51%	2.10	0.15
Other	0.90%	0.00%	0.66	0.42

Bold indiactes significant value (*p* < .05)

The most often mentioned theme for blended learners was that they could not think of any improvements, while 20% of online learners mentioned this, blended learners mentioned it significantly more often. For example, “no improvements necessary, live chat is great, approachable, responsive and met my needs”.

One-quarter of learners made a comments about availability. Common within this theme was the request that live chat should be available more often “I'd like to see it available more frequently” and “I'm not 100% sure if it is available on weekends but that would be awesome”, and that there should be more staff using it “making more tutors use it—or more availability to use it more often”. Even so, while they wanted it available more often, some learners realised staff cannot be available 24/7 “Possibly having it online more often, however this may be unrealistic, as teachers have lives” “Understandably, you're all very busy. But more live chat access!”.

Even though when live chat was available it popped up on the home screen of the subject’s site on the LMS when staff were available during virtual office hours, some learners wanted more set times and more notification when staff were available. For example, “Students should be aware of certain times that live chat is available” and “Have them more often or send out emails when they are online so that I am aware of when I can ask a question”.

Approximately one-quarter of learners suggested potential improvements to design. These included wondering if it could be made anonymous. While live chat allows the user to send documents and to save the conversation, some students requested both of these features as improvements to design. “Perhaps provide a way to generate an email copy of the chat text to be available to the student”. Others suggested an online whiteboard or ability to video call during chat “Maybe include a board where you can upload illustrated examples of the concept your trying to understand, and create a drawing tool to lectures can aid their answers visually”.

### Other methods of communication used for help-seeking by blended and online learners

#### When seeking help why they choose to use live chat

Table 7 shows that six themes emerged plus an ‘other’ theme regarding the reason they looked for help on live chat. The majority of participants stated it was because it was urgent or they needed a quick response. Chi-square analyses showed that online learners mentioned it was for privacy reasons significantly more often than blended learners. Themes are discussed in more detail below.

**Table 7:** Reasons online and blended learners used: live chat

The reason I asked my question on live chat was…	Proportion of blended learners *n* = *136*	Proportion of online learners *n* = *80*	Chi-square	*p* value
I wanted an urgent / quick response	63.97%	75.00%	2.82	0.09
I needed general / unspecified help	23.53%	13.75%	3.02	0.08
I needed assessment help	18.38%	13.75%	0.78	0.37
For privacy	6.62%	15.00%	4.03	**0.04**
For support / embarrassed	4.41%	3.75%	0.06	0.81
I was not on-campus	2.94%	3.75%	0.11	0.74
Other	0.74%	2.50%	1.15	0.28

Bold indiactes significant value (*p* < .05)

The majority of both blended and online learners said they chose live chat as their method of communication when they wanted a quick response from staff. For example “For urgent questions or If I was in the process of working on an assignment or homework task and I needed help or clarification or wanted to get an immediate back and forth dialog going about a subject that interested me”.

The next two most common themes related to the types of questions the learners asked. The biggest group were general questions or questions where the content of the question was not specified. The other group of questions related to their assessment e.g. “For quick responses to questions I come across as I work through my assignment”.

A small portion of students commented about getting support or feeling embarrassed to ask for help through other methods e.g. “being my first [subject/course] at University I didn't want to sound stupid asking questions that may be obvious to others”. In a similar vein, some students chose this method of communication because it was perceived as being private e.g. “Its private and fast. I love that you get a quick response and you don't need to carefully word your question to prevent other students from taking your ideas”. This was significantly more important for online learners than blended learning students.

#### What reason do you use email

Table 8 shows that seven themes emerged plus an ‘other’ theme regarding the reason they looked for help using email. The majority of participants stated their use of email was because their questions were of a private or formal nature. Themes are discussed in more detail below.

**Table 8:** Reasons online and blended learners used: email

The reason I asked my question on email was…	Proportion of blended learners *n* = *139*	Proportion of online learners *n* = *83*	Chi-square	*p* value
It was private / formal	55.40%	66.27%	2.55	0.11
I needed assessment help e.g. extensions, grades, feedback, resubmission	25.90%	31.33%	0.76	0.38
I had a long detailed question	15.11%	18.07%	0.34	0.56
I want to talk to a specific person	15.11%	9.64%	1.37	0.24
I wanted a record	3.60%	7.23%	1.39	0.23
It was not urgent	5.76%	8.43%	0.59	0.44
Live chat was not available	5.04%	2.41%	0.54	0.46
Other	4.32%	7.23%	0.86	0.35

The most common theme was that they used email for anything private or formal. Most students did not elaborate further on the personal reason that they felt necessitated a private means of exchange with teaching staff.

Along similar lines, the next biggest theme emerging from student responses about using email were questions related to a grade on an assessment, feedback or an wanted an extension for assessment. Most of the responses pertained to requiring extra time for an assignment “Asking for extensions or consideration.”

Learners mentioned they use email for complex questions, long questions or where they expected a detailed response: “If it is likely to be a long question or I need to think out the answer slowly, or if I have many things going on that day so I can add to this question”. They also used email if they wanted to talk to a specific person e.g. “for when I have a question that is directed at a certain individual (e.g. [subject co-ordinator], seminar teacher)”.

For a smaller proportion of learners, they used email if they wanted to keep a record of the conversation “for traceability (evidence), that the exchange took place”, or if it was not urgent or live chat was not available.

#### What reason do you use discussion boards?

Table 9 shows that six themes emerged plus an ‘other’ theme regarding the reason they looked for help using the discussion boards. The majority of participants stated it was because of collaborative learning. Chi-square analysis showed that online students were more likely to mention using discussion boards when no privacy was required. Themes are discussed in more detail below.

**Table 9:** Reasons online and blended learners used: discussion boards

The reason I asked my question on discussion boards was…	Proportion of blended learners *n* = *139*	Proportion of online learners *n* = *81*	Chi-square	*p* value
For collaborative learning (e.g. benefit from or to others)	60.43%	67.90%	1.23	0.27
I needed general help	30.22%	38.27%	1.50	0.22
Assignment help	26.62%	17.28%	2.50	0.11
I did not need an urgent response	4.32%	11.11%	1.50	0.22
Live chat was not available	2.16%	3.70%	0.46	0.49
No privacy was needed	1.44%	8.64%	6.77	**0.01**
Other	1.44%	3.70%	1.18	0.28

Bold indiactes significant value (*p* < .05)

The common reason for using the discussion boards was for collaborative learning. Students used this method when they thought the question and responses would benefit others, or that they would benefit from calibrating their understanding against their peers. For example, “posting questions which are likely to be concerns of others as well—so teaching staff only have to respond once” or “for questions that I would like other students’ opinions on e.g. other ways of seeing the same information to gain a better understanding” or “to obtain differing viewpoints on a topic where multiple opinions are useful or valid”.

The types of questions students asked were related general questions e.g. “general questions regarding content, referencing, assignments, classes etc.” Again this often related to anyone being able to respond and being useful to others. Students mentioned that these questions were unlikely to require privacy, or that the students were “not embarrassed asking”. Students also mentioned that they used the discussion boards when live chat was not available or help was not urgently needed.

## Discussion

The current study aimed to explore the use of live chat technology for online academic help-seeking within higher education subjects, with a particular interest in exploring any differences related to study mode as an online or blended learning student. Both blended learning students and online students were overwhelmingly satisfied with the use of live chat as a tool for online academic help-seeking. Notably, they felt that by using live chat, the teaching team cared about supporting their learning, and students found it useful for just-in-time support, and would recommend it to other learners. These findings align with other studies that have found that students have high levels of student satisfaction (e.g. Luo et al. 2017; Robles et al. 2019), like that responses are immediate and timely (Lauricella & Kay, 2013), and feel an enhanced sense of connection (Klien et al. 2016; McInnerney & Roberts, 2004) when using instant messaging tools.

As predicted, online learners at times did significantly differ in their perceptions of live chat compared from blended learners. While all learners endorsed the use of live chat as a help-seeking tool, online learners were significantly more positive than blended learners, highlighting the importance of such a tool for these students. This is a particularly critical issue for online students, who can feel isolated from peers and the institution given they are not on campus. From the thematic analysis, two reasons that were mentioned significantly more often by online students stand out as to why online learners may have preferred live chat more than blended learners. The first is the ability to access teaching staff, and to be able to approximate face-to-face interaction they miss out on as an online student; and second the ability to get a quick response and just-in-time help from staff. As the usual modes of communication for online students in this context were online discussion boards and email, it is unsurprising that these are two important themes for them. These comments demonstrate the importance of real-time connection with teaching staff, teacher presence, and being able to get help in-the-moment rather than waiting for a delayed response for online students. These findings also contribute further understanding of help-seeking behaviours of different students groups. Previous research has found that help-seeking is often an underutilised SRL strategy for online learners (Broadbent, 2017; Chen, 2002; Puzziferro, 2008). But possibly, measures of online help-seeking do not adequately capture online help-seeking, or online students have not been given satisfactory means to help-seek online. The current findings suggest that, with the right tool, online students do embrace online help-seeking strategies.

Overall, all students liked that live chat was an instant, real-time help-seeking tool that was convenient to use. They said that they found live chat most useful when they needed an urgent response, particularly around assessment time and mostly asked questions—unsurprisingly—about their assignments. Interestingly, while all students found live chat useful to get clarification around assessment, the blended learning students were significantly less likely to ask questions related to asking for extra time to complete assessment, discussing the grade and feedback on their assessment or asking for the assessment piece to be re-marked. This did not appear to be problematic for online students, perhaps because of their limited means for help-seeking, but online students did often mention this was a reason they would preference the use of email. Overall, however, both groups of students said they were comfortable asking anything on live chat, they thought no improvements were needed, except that it should be available more often and in more subjects.

When asked about alternative help-seeking methods, all students said they were likely to use email for more formal personal and private matters. While live chat was private, from the students’ responses, it appears that email was thought to be more private, perhaps because the student understood who would be responding to their question and could direct their question more specifically to a single person. Also, email was preferred, as previously mentioned, when students wanted to dispute a grade, feedback or get a remark, which may have required some thought about how to put together the question. This finding aligns with another theme that email was preferred if they had a long and detailed question, aligning with previous studies looking at student communication preferences (Lodge, 2010). When asked about discussion boards, students overwhelmingly said they used when they thought that their question (and its response) would be of benefit to other learners or that they would benefit from calibrating their understanding against their peers. This result also fits the findings of other researchers (e.g. Kitsantas & Chow, 2007) and shows that learners do make decisions about how best to ask questions, which are not based solely on their own needs.

The current study used live chat in eight psychology subjects where the bulk of the students were female psychology students, live chat may be particularly suitable for this segment of the broader higher education population. Exploration of other courses, particularly male-dominated courses, would be useful in future research. Further, it is not known what was discussed between teachers and students on live chat, only what students reported in the survey, which may have been different if transcripts had been analysed. Gathering this information may give some useful insights into what types of questions, time of day live chat was most frequently used, and time required to use the tool.

Lastly, this study did not acquire perceptions of using live chat from teaching staff; this, would be useful to explore in future research. While this study utilised live chat during pre-arranged office hours chosen by the teaching staff, Li and Pitts (2009) have shown that virtual office hours increase both perceived workload of staff and student expectation of teacher access. Few studies have explored teachers' perceptions, concerns and challenges around using instant messaging services (Tang & Hew, 2017). Tang and Hew (2017) discussed that teachers need guidance on how to use instant message services efficiently and when it is most beneficial use for both teachers and students. While ensuring that online students are supported and have the same ability to connect synchronously with teaching staff is important, teaching staff, particularly those juggling competing responsibilities, need to feel comfortable using this type of technology for communication. Being instantaneously available for live chats with students has potential downsides for teaching staff, such as leaning towards a service provider model of education, encouraging student dependence and potentially further promoting poor-work life balance by adding another thing for academics to do. While a full discussion on teachers perceptions of using live chat is outside the scope of this study, these are potential limitations to using live chat as a communication tool. Further explorations of teachers perceptions are warranted.

### Conclusion and implications

The implications of this study is that it has demonstrated the efficacy of live chat as an online help-seeking tool in higher education between teacher and student. The tool was well received by both online and blended learners, particularly its ability to provide instant, real-time, and convenient help. Live chat was particularly well received by online learners, who were more satisfied, felt more cared about by the teaching team and would recommend it to others. Further, online learners mentioned that they like that it gave them better access to staff and gave them the ability to have their version of face-to-face conversations. As online, synchronous, private help-seeking tool between student and teacher, these findings provide a strong recommendation for the use of live chat in higher education.

## Data Availability

Not available.
